# Paediatric Salmonellosis—Differences between Tropical and Sub-Tropical Regions of Queensland, Australia

**DOI:** 10.3390/tropicalmed4020061

**Published:** 2019-04-10

**Authors:** Daria Berger, Felicity Smith, Vana Sabesan, Aimee Huynh, Robert Norton

**Affiliations:** 1 Department of Paediatrics, Townsville Hospital, Townsville 4814, Australia; daria.romanik@gmail.com (D.B.); Vanaja.Sabesan@health.qld.gov.au (V.S.); huynh.aimee@gmail.com (A.H.); 2 College of Public Health and Tropical Medicine, James Cook University, Townsville 4814 Australia; felicity.smith1@jcu.edu.au; 3 Department of Microbiology and Pathology, Townsville Hospital, Townsville 4814, Australia

**Keywords:** paediatric, Salmonella, Australia, tropical

## Abstract

Salmonellosis is an important cause of morbidity in tropical regions.This study aims to describe the epidemiology of non-typhoidal *Salmonellae* (NTS) in children presenting to public hospitals in Queensland, Australia, over the past 20 years, with a focus on differences between tropical and sub-tropical zones in the region. This is a retrospective and descriptive cohort study of 8162 NTS positive samples collected in 0–17-year-olds from the Queensland public hospital pathology database (Auslab) over a 20-year period from 1997 to 2016. There were 2951 (36.2%) positive NTS samples collected in tropical zones and 5211 (63.8%) in the sub-tropical zones of Queensland, with a total of 8162 over the region. The tropical zone contributed a disproportionately higher number of positive NTS samples by population sub-analysis. Of the specimens collected, 7421 (90.92%) were faecal, 505 (6.2%) blood, 161 (1.97%) urine, 13 (0.16%) cerebrospinal fluid (CSF) and 62 of other origin. Other categories of specimen types isolated include swab, fluid, aspirate, lavage, bone, tissue, isolate and pus, and these were not included in sub-analysis. The most commonly identified serovars were *Salmonella* Typhimurium, *Salmonella* Virchow and *Salmonella* Saintpaul. This is the first and largest study that emphasises the high burden of invasive and non-invasive NTS infections resulting in hospital presentations in the paediatric population of tropical north Queensland, compared to the sub-tropics.

## 1. Introduction

*Salmonellae* are motile, gram-negative, facultatively anaerobic bacilli that can be serotyped into more than 2500 serovars. These include *Salmonella* Typhi and the paratyphoidals Salmonellae which are synonymous with “typhoid” and “enteric fever”. These caused 216,500 deaths in 2004 in Asia alone [1,2]. Exact mortality figures vary widely due to limited surveillance in highly endemic regions [2,3,4] and specific paediatric data are limited. In Australia, *S.* Typhi and *S.* Paratyphi are predominantly imported from travellers [5]. Globally, non-typhoid *Salmonella* (NTS) are becoming evident as a major cause of paediatric bacteraemia in areas such as Sub-Saharan Africa where malaria, HIV and malnutrition contribute a higher rate of mortality [6,7,8,9,10]. It has been estimated that >600,000 deaths are caused by NTS diarrhoeal disease per year across the world [11,12].

In Australia salmonellosis is a notifiable disease, and in 2014 the National Notifiable Disease Surveillance System (NNDSS) identified a total of 16,358 cases of non-typhoid salmonellosis, which is the highest number recorded since 1991 [13]. Notification rates were highest in the Northern Territory at 186.8 per 100,000 population and lowest in Tasmania at 48.4 per 100,000 population. The highest frequency was noted in the 0–4 year age group at 23%, with an age-specific rate of 210.4 per 100,000 population compared to 69.7 per 100,000 for the whole population [13].

A recent study on paediatric bacteraemia by Er et al. found higher rates of salmonellae isolated from blood cultures in North Queensland compared with other parts of Australia [14].

Non-typhoid *Salmonella* spp. are predominantly food-borne [15], and the incubation period varies from 6 to 72 h, although it can be as long as 16 days. A temporary carrier state lasting more than a year has been described, especially in infants [16]. Symptoms are generally mild and consist of vomiting, diarrhoea and fever, although people who are immunosuppressed and at the extremes of age appear more likely to develop invasive disease [9,17,18]. Although *S.* Typhi is solely human in its reservoir, most NTS serovars display a complex relationship between animals and their environments, such as water and soil [19]. Ecological studies have proposed that the genomic adaptability of *Salmonella* spp. reflects their ubiquitous presence in the environment [20].Research has also demonstrated NTS antibiotic resistance to: ampicillin, tetracyclines, third generation cephalosporins and quinolones in countries with tropical regions including: Zambia, Kenya and China [21,22,23,24].

This study was done to determine whether there is a difference, and to define that difference, in Paediatric *Salmonellosis* (invasive and non-invasive), between defined tropical and sub-tropical regions of Queensland. The importance of this would be in informing public health, clinicians and the public of risk groups for this common yet potentially fatal condition.

## 2. Methods

Retrospective data were sourced over a 20-year period between 1997 and 2016. Inclusion criteria for analysis were: specimens of faeces, blood or CSF that were either culture or nucleic acid amplification testing (NAAT) positive for salmonella; age 0–17 years; specimen collected between 1997 and 2016; and specimen processed in a Queensland Health facility. 

**Figure 1:**
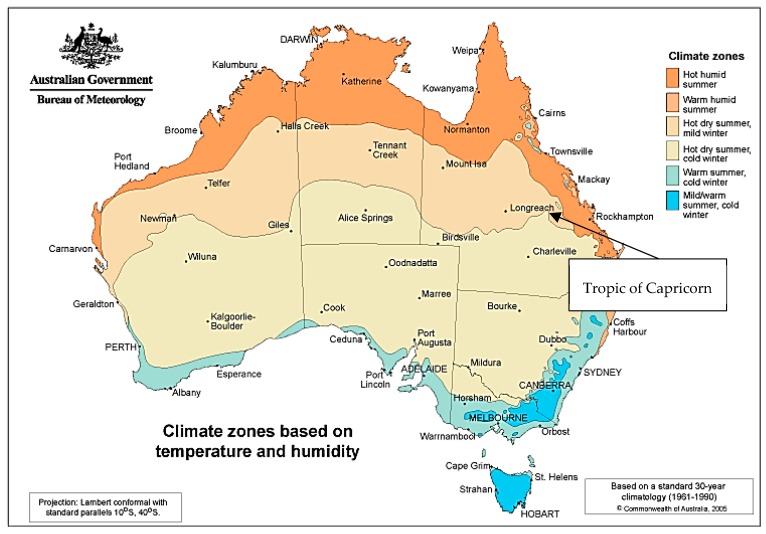
This describes climactic zones. Zone 1 (dark orange, tropics), which is entirely in the tropical north, is defined by high humidity summer and warm winter, zone 2 (light orange, sub-tropics) is defined by warm humid summers and mild winters, and zone 3 (yellow, sub-tropics) is defined by hot dry summers and warm winters. (Australian Government, Bureau of Meteorology, Climate Classification Map).

Exclusion criteria were: age and year of collection outside the accepted range, other pathogens, repeatedly positive samples for the same specimen with the same organism, no collection time specified due to possible collection error, and samples not identified as corresponding to Queensland Health facilities. Duplicate samples for the same specimen type and individual were not included. Data were accessed from Auslab, which is a Pathology Queensland database representing public pathology across Queensland. Collected variables included: sex, date of birth, age of patient in months, date of collection, specimen type, species of salmonella including antibiotic susceptibilities, and location at which the sample was collected. Location was further divided into tropical and sub-tropical regions based on Queensland Health service districts. Districts that lie mostly above the Tropic of Capricorn, and which are thus within a tropical climate, were labelled tropical, and all others as sub-tropical. Referring to the Tropic of Capricorn to define tropical and sub-tropical regions geographically is in keeping with the definition used in the general scientific community. Figure 1 shows the division between the regions described.

Detailed geographic distribution of population from the Australian Bureau of Statistics (ABS) was available only for the year 2011. Although it would clearly be ideal for this study to use an annual distribution of population for the remaining years, this was not available. The regions studied were divided into tropical and sub-tropical areas based on the local government area (LGA) that most closely overlapped with the Queensland Health service district. This 2011 population distribution was used to calculate incidence rates of cases across regions, which were compared using a Chi-square test. Population distribution data for other years was not available.

Data were organised for frequency analysis by age, age group, sex, specimen, serovar, postcode, year collected, and district.

The isolation of faecal pathogens was done directly from clinical samples of faeces, blood, urine or pus. In the isolation of Salmonellae from faecal samples, these were cultured on XLD agar (Biomérieux, Marcy l’Etoile, France) and incubated at 37 °C in air for 48 h. Non-lactose fermenting or H_2_S-producing colonies were selected for identification either by the Vitek 2 or MALDI-TOF (Biomérieux, Marcy l’Etoile, France). Samples of faeces were also inoculated into Selenite broth for enrichment prior to subculture onto XLD agar (Biomérieux, Marcy l’Etoile, France). Potential colonies of Salmonellae were further identified as above.

More recently there has been a change to NAAT of faeces for gastrointestinal pathogens. Any that were positive for Salmonella were reflex cultured as above and confirmed. These were then also available for serotyping. Non-faecal specimens were cultured using standard methods and identified as above.

Antibiotic susceptibility was determined by the methods of The Clinical and Laboratory Standards Institute (CLSI) [25] or those of the European Committee on Antimicrobial Susceptibility Testing (EUCAST) [26] for trimethoprim-sulfamethoxazole and ampicillin. This was done using disk diffusion as described. Mean age with standard deviation of cases was calculated for tropical and sub-tropical regions. The proportions of faecal, blood and cerebrospinal fluid (CSF) specimens collected in both regions were calculated and compared. Further data were sourced from the Queensland Communicable Disease Branch (CDB) regarding total salmonella notification rates throughout the state per age group of 0–17 years from 1996 to 2016. This CDB data was used in conjunction with ABS data on Queensland age groups to compare the frequency of notification of Salmonellosis by age group during the study period. Data were analysed using SPSS and Windows Excel. Ethics approval was granted by the Townsville Health Service District Human Ethics Committee (HREC/16/QTHS/105).

## 3. Results

**Figure 2:**
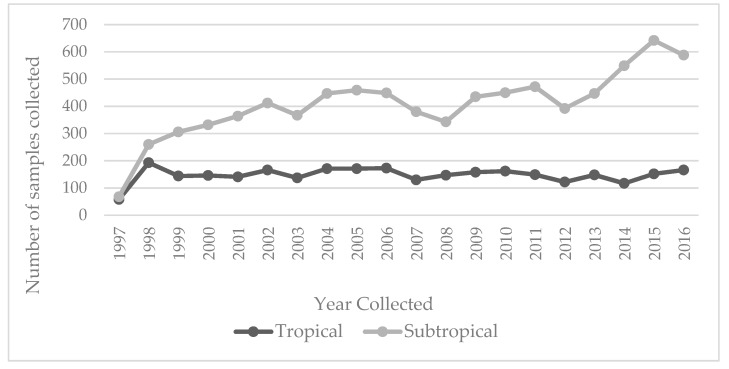
Total number of non-typhoid *Salmonella* (NTS) positive specimens per region per year collected.

There were 8162 positive specimen samples that were included in the analysis. Of these, 2951 (36.2%) of samples originated from the tropical region and 5211 (63.8%) from the sub-tropical region. Figure 2 presents the number of samples collected per region per year.

In 2011, the year for which detailed regional ABS population data are available, the incidence of paediatric salmonella requiring a public hospital visit was significantly greater in the tropical compared to the sub-tropical region (84.2 per 100,000 tropical, 36.1 per 100,000 sub-tropical (Chi-square test: χ^2^ = 77.652, df = 1, *p* < 0.001)). Statistical analysis using a Chi-square test could be performed only for the 2011 population sub-analysis due to data access issue. Consequently, further comments comparing sites is descriptive.

Of the 8162 positive specimens, 7421 (90.92%) were faecal, 505 (6.19%) blood, 161 (1.97%) urine and 13 (0.16%) cerebrospinal fluid (CSF). Other specimen types included swab, fluid, aspirate, lavage, bone, tissue, isolate and pus, and these contributed 0.75% of all positive samples. These were not included in specimen sub-analysis.

### 3.1. Age Distribution

**Figure 3:**
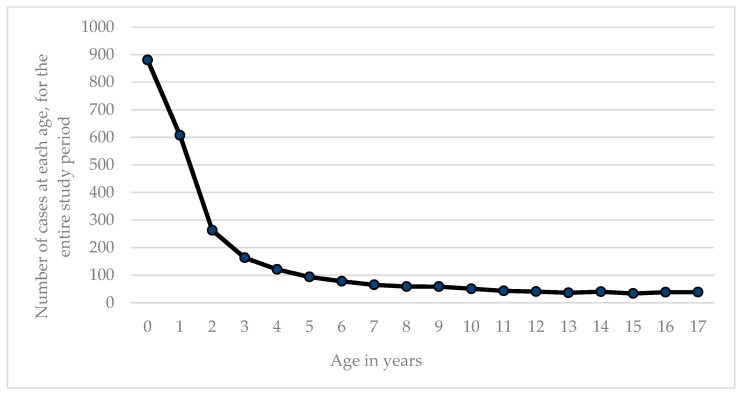
of NTS salmonellosis in Queensland over the period 1997–2015 by age in years from Salmonellosis notification and ABS population data.

The total population of children aged 0–17 years in Queensland increased steadily over time from 1997 to 2015 with an initial population of 871,548 increasing to 1,127,278. The total frequency of salmonella notifications in the paediatric population in Queensland from 1997 to 2015 was 35.9 per 100,000 children. The number of positive specimen samples per age group decreased with increasing child age. This data are represented in Figure 3.

The mean age of the affected population of the northern region was significantly less than the southern (t = −12.12, df = 7200.86, *p* < 0.001), with a mean age of 27.5 months (SD = 39.83) compared with 39.6 months (SD = 49.15), respectively. Sex distribution was similar across both regions (males tropical, 54.3%; males sub-tropical 53.8% (Chi-square test: χ^2^ = 0.209, df = 1, *p*=0.647)).

### 3.2. Faecal Specimens

Of the 7421 positive faecal samples, which represented non-invasive disease, 36.53% were isolated from tropical regions compared with 63.47% from the sub-tropical regions. This did not reflect the population proportions between the two groups (16.49% tropics and 83.51% sub-tropics). Of all positive samples collected in the tropical region, 91.87% were faecal compared to 90.39% of all those collected in the sub-tropical region. The highest number of positive faecal samples collected was in the under 12-month age group. This pattern was seen in both regions. 

### 3.3. Blood Cultures

Of the 505 positive blood cultures, which represented invasive disease, 31.68% were from the tropical regions compared with 68.32% from the sub-tropics. Of all the positive blood cultures collected in the tropical region 5.42% were blood, and of all those collected in the sub-tropical region 6.62% were blood. As with positive faecal specimens, the highest number of positive blood cultures was seen in the under 12-month-age group. This pattern was also similar across both regions. There was a total of 141 positive blood samples identified in the <12 month age group over the data-collection period, of which 43 (30.5%) were identified in the tropics and 98 (69.5%) in the sub-tropics.

### 3.4. Cerebrospinal Fluid (CSF)

The total number of positive CSF samples over the 20-year period was 13, of which 8 were collected in the sub-tropical region. All CSF samples were collected in infantsaged under 9 months. These numbers were too small to make any meaningful interpretation in differences between regions.

### 3.5. Serovar Distribution

**Figure 4:**
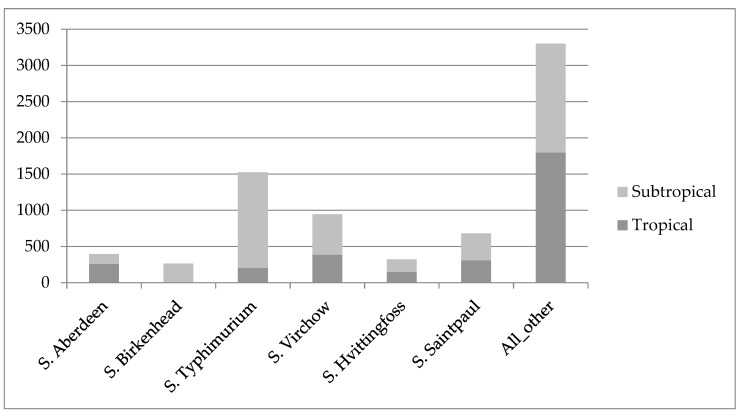
Distribution of faecal *Salmonella* spp. serovars in the tropical and the sub-tropical regions.

The number of different salmonella serovars identified was 106, with a total of 776 positive specimens that were untypable. The differences in serovar distribution between the two regions are shown in Figure 4. Proportional distribution of the most common serovar, *S*. Typhimurium, was found to vary significantly between tropical and sub-tropical regions, consisting of 7.5% of total tropical paediatric cases, and 25.7% of sub-tropical paediatric cases (Chi-square test: χ^2^ = 408.16, df = 1, *p* < 0.001).

The four most common serovars across the entire data set were *S*. Typhimurium, *S*. Virchow, *S*. Saintpaul and *S*. Aberdeen, and this pattern was the same in both tropical and sub-tropical regions. The most commonly isolated serovars in the 0–4-year age group were: *S.* Virchow (15.6%), *S.* Typhimurium (13.1%), untypable (9.9%), *S.* Saintpaul (8.4%), *S.* Aberdeen (6.7%), and *S*. Waycross (4.4%). Appendix A provides detailsofSalmonella serovars identified in the data set. 

There were few cases of *S*. Ball and *S*. Urbana, with six cases of *S*. Urbana found only in tropical areas, 2 cases of *S*. Ball in tropical, and 2 cases of *S*. Ball in sub-tropical areas.Within this age group, males made up 53.3% of cases andfemales made up the remaining 46.7%. 

There were 24 cases of *S.* Typhi, 8 of *S.* Paratyphi A, and 6 of *S.* Paratyphi B during the data-collection period. As these were likely imported cases, they were not included in further analysis.

### 3.6. Antibiotic Susceptibility Testing

Across both regions, most *Salmonella* spp.isolates tested were susceptible to the two antibiotics primarily used in the treatment of salmonellosis (trimethoprim/sulfamethoxazole and ampicillin). A summary of this is provided in Table 1.

## 4. Discussion

This is the first and largest study to describe the epidemiology of paediatric salmonellosis across Queensland, Australia over a 20-year period with a focus on tropical and sub-tropical regions. The greater incidence of paediatric salmonellosis cases in the tropical region is statistically significant (2011 incidence 84.2 per 100,000 tropical, 36.1 per 100,000 sub-tropical (χ^2^ = 77.652, df = 1, *p* < 0.001)). This demonstrates a higher burden of NTS infection in the tropical region of Queensland. The greatest number of cases of paediatric salmonellosis was observed in the under 12-month age group, a finding which is supported by other recent epidemiological studies [27].

Although the overall number of children in Queensland increased steadily over the observation period, the number of public hospital salmonella cases per area per year did not follow the same pattern. The sharp increase in cases in the sub-tropics in 2015 may reflect a shift in laboratory identification techniques from traditional culture to NAAT techniques, which are more sensitive [28], and this change in technique may bias results. However, Kirk et al. described an increasing trend for salmonellosis since 2010 adjusted for population growth [15].

As approximately 71% of salmonella cases are food-borne [29], factors such as inability to maintain food hygiene practices, poor sanitation and overcrowding may contribute to the higher incidence of salmonellosis in tropical Queensland. Additionally, multivariable analysis conducted by Gibney et al. has highlighted the fact that the notification incidence for salmonellosis over a 21-year period was three times higher for residents of remote or very remote areas, with a disparity in notification incidence in Indigenous Australians with no significant difference based on socioeconomic disadvantage [30]. Socioeconomic differences across regions have also been shown in previous studies not to influence food hygiene practices [31,32].

Climatic differences may partly explain the finding of differences across tropical and sub-tropical Queensland. “Warm and wet” regions such as North Queensland have been shown to have the highest rates of salmonellosis [33], and an increase in temperature and rainfall has been correlated with an increase in cases [34]. The inclusion of region-specific temperature and rainfall data is beyond the scope of this study. Fearnley et al. have previously demonstrated, using Bayersian source attribution models, that Queensland has higher reported rates of NTS salmonella than other temperate states of Australia and that environmental factors may play a greater role in transmission in younger children rather than specific reservoirs such as egg, chicken, and nuts [35].This opposes previous suggestions by Messer et al. that attributed cases of salmonella meningitis in Cairns to gaps in poultry and egg processing [36]. A link has also been demonstrated between *S.* Virchow and the Asian house gecko in houses in northern Queensland [37]. Additionally, Williams et al. demonstrated the importance of frogs, dogs and vacuum dust as a reservoir for faecal-oral transmission in children aged 0–4 years in the Northern Territory [32]. The importance of pet reptiles as a source of NTS species causing invasive disease has also been shown [38,39,40], as has proximity to contaminated bore water, river water, dust, and food industry-related packing sheds [29,41]. Due to its apparent ubiquitous nature, additional factors to consider in variations in salmonella transmission include inoculating dose, the role of immunity and human-to-human transmission in perpetuating the disease, and known risk factors such as antibiotic use or the use of pH-reducing medications [42,43].

The observation that younger age groups were significantly more affected than older age groups may be related to immunological factors, developmental stages, and associated lack of hygiene awareness. Additionally, typical serovars seen in food-borne disease may not be expected to be seen in the youngest children (0–3 months) that are not yet eating solids. Contrary to this, *S.* Typhimurium, which has been associated with contaminated egg outbreaks [44], was observed as a common pathogen in the sub-tropical region in this age group. This may reflect spread through asymptomatic shedding or faecal-oral spread from affected contacts. The finding of *S.* Typhimurium as an important pathogen as a well as the high rate of *S.* Virchow, *S.* Saintpaul, *S.* Birkenhead, *S.* Aberdeen, and *S.* Hvittingfoss in Queensland is supported by a recent study by Ford et al. [27]. The finding of *S.* Virchow as an important pathogen has previously been described by Ashdown et al. [45]. There were no specimens with dual isolates of different Salmonella serovars.

There was no difference in susceptibilities of isolates from the two regions to two of the antimicrobials tested (trimethoprim/sulfamethoxazole and ampicillin). Third-generation cephalosporin susceptibility was not tested as most isolates were from faeces. Susceptibility testing was not uniform and, as such, conclusions on resistance trends are difficult to make.Antibiotic therapy is not indicated for the large majority of cases of gastroenteritis due to *Salmonellae*. As such, only a limited number of antibiotics are tested and reported for non-invasive disease, which is the majority in this report. Quinolones in particular are not recommended for non-invasive disease and as such are not reported here. This is in accordance with the Australian Therapeutic Guidelines [46].

This study demonstrated that central nervous system involvement only affected children under 9 months of age. Although numbers are small, 38.5% (5 cases) were from the northern region, despite this region only having 16.49% of the population. A previous study on *Salmonella* meningitis did not establish individual factors influencing this.

There are several limitations to this study. These are largely due to the retrospective nature of the data analysed and the lack of standardisation of data collection. Population bias is likely to influence results through discrepancies in ABS local government area numbers compared to hospital district data, and as these areas do not overlap exactly, there will likely be a margin of error in 2011 sub-population comparison and Chi-square test. The inability to obtain annual population distribution data for years other than 2011 is another significant limitation. Direct comparison of the incidence between tropical and sub-tropical areas of Queensland was not performed due to lack of access to specific salmonella notification numbers per population from the public-health unit. Annual incidences of salmonella presentations were unable to be calculated due to lack of population data by region and age. Similarly, comments of Indigenous versus non-Indigenous findings could not be described. Additionally, variability in time periods and salmonella notification methods makes this data difficult to compare. Some conclusions were drawn on the assumption that paediatric population proportions remained relatively constant between regions during the study period. Discrepancies between inclusion and exclusion criteria used by the surveillance network in comparison to the criteria used in this study will also bias findings. Surveillance data also lends itself to bias due to possible inaccuracies in collection of data and analysis. There was no access available to private pathology data. However, public health salmonella notification data are considered to be an accurate reflection of actual cases. It should also be noted that reinfections would have been missed, as repeat samples from the same patient were excluded.

The strength of this study lies in its ability to demonstrate disease patterns and population factors across a wide time frame, geographical location, and a large population base. Our findings suggest a significantly higher hospital burden of salmonella-related disease in the tropical north of Queensland compared to the sub-tropical south. The salmonella burden is highest in the 0–4-year age groups. 

To our knowledge, this is the first Australian study that shows the contribution of NTS to meningitis in the under 9-month age group. This is also overrepresented in the tropical region. An emphasis on education in the relevant health districts for public health promotion regarding food preparation and hand hygiene, targeting families with young children, would be beneficial. Additionally, this study highlights the need for a national, unified, and paediatric-specific salmonella surveillance with a focus on tropical regions.

## Figures and Tables

**Table 1:** susceptibility in tropical and sub-tropical regions for all positive specimens collected (both European Committee on Antimicrobial Susceptibility Testing (EUCAST) and The Clinical and Laboratory Standards Institute(CLSI) methods used).

Antibiotic	Tropical Region			Sub-Tropical Region		
	Number Susceptible	Number Tested	Percentage Susceptible (95% C.I.)	Number Susceptible	Number Tested	Percentage Susceptible (95% C.I.)
Ampicillin	2579	2653	97.21 (96.58,97.84)	4322	4544	95.11 (94.49, 95.74)
Trimethoprim/Sulfamethoxazole	2539	2563	99.06 (98.69, 99.44)	4350	4458	97.58 (97.13, 98.03)

## Appendix A

**Table A1:** Frequencies of Salmonella Serovars in tropical and sub-tropical areas for all positive samples.

Serovar	Tropical	Sub-tropical	Total
Aberdeen	147	310	457
Abony	3	0	3
Adelaide	7	29	36
Agona	17	49	66
Amsterdam	1	0	1
Anatum	52	28	80
Arizonae	6	2	8
Bahrenfeld	6	1	7
Ball	2	2	4
Bareilly	0	3	3
Beaudesert	1	0	1
Bergedorf	1	0	1
Birkenhead	2	274	276
Blockley	0	1	1
Bovismorbificans	18	36	54
Braenderup	3	1	4
Brazzaville	0	1	1
Bredeney	8	14	22
Breukelen	3	0	3
Bukavu	1	1	2
Cerro	1	4	5
Chailey	30	9	39
Charity	0	2	2
Chester	112	145	257
Concord	0	1	1
Corvallis	5	25	30
Dan	2	0	2
Derby	1	0	1
Eastbourne	12	14	26
Emek	1	0	1
Emmastad	2	1	3
Enteritidis	73	50	123
Falkensee	1	0	1
Ferruch	0	1	1
Give	4	5	9
Hadar	1	0	1
Havana	12	20	32
Heidelberg	62	55	117
Hessarek	0	1	1
Hvittingfoss	173	154	327
Infantis	8	29	37
Isangi	0	1	1
Java	2	0	2
Javiana	0	42	42
Johannesburg	7	3	10
Kentucky	0	2	2
Kiambu	4	26	30
Kimberley	1	0	1
Kinondoni	1	0	1
Kottbus	6	8	14
Lansing	18	7	25
Lindern	1	0	1
Litchfield	20	33	53
Livingstone	0	1	1
Mbandaka	6	9	15
Meleagridis	1	0	1
Mgulani	101	24	125
Mikawasima	0	2	2
Mississippi	0	5	5
Montevideo	19	20	39
Muenchen	47	149	196
Muenster	0	1	1
Mugulani	1	0	1
Napoli	0	1	1
Newport	3	14	17
Ohio	1	2	3
Ohlstedt	8	9	17
Onderstepoort	1	2	3
Oranienburg	10	8	18
Orientalis	14	38	52
Orion	38	5	43
Oslo	4	7	11
Panama	0	7	7
Para B Biovar Java	44	56	100
Paratyphi A	1	7	8
Paratyphi B	4	2	6
Poona	9	5	14
Potsdam	3	61	64
Ramatgam	1	0	1
Reading	54	49	103
Rissen	0	1	1
Rubislaw	4	12	16
Saintpaul	327	399	726
Salford	0	1	1
Sandiego	14	3	17
Schwarzengrund	1	2	3
Senftenberg	1	0	1
Singapore	5	18	23
Stanley	7	12	19
Tennessee	2	3	5
Thompson	36	4	40
Typhi	15	9	24
Typhimurium	222	1348	1570
Uganda	6	0	6
Untyped	307	469	776
Urbana	6	0	6
Virchow	460	704	1164
Virginia	1	1	2
Wandsworth	11	1	12
Wangata	1	28	29
Warneri	1	0	1
Waycross	63	246	309
Welikade	143	5	148
Weltevreden	70	24	94
Yarrabah	1	0	1
Zanzibar	37	45	82
Zehlendorf	2	2	4
Total	2951	5211	8162
